# Mundesley Sanatorium for Consumption

**Published:** 1905-06-17

**Authors:** 


### MUNDESLEY SANATORIUM FOR CONSUMPTION.

IT has been shown by Dr. Burton Fanning and others that the climate of part of East Anglia is particularly suitable for the open-air treatment of consumptive patients. The rainfall is low; there is a great deal of sunshine; very little, if any, fog, and the air is pure and bracing. The soil is of a sandy nature and is soon dry even after very heavy rains. From Liverpool Street, Mundesley may be reached after a run of five hours, and from King's Cross a little longer must be allowed. The Sanatorium, which is in the parish of Gimingham, stands on a site of 20 acres in extent, and is about 15 minutes' walk from the Mundesley Station. Cromer is about six miles off.

The position of the building has been very well chosen. It is at the foot of a pine-clad hill which shelters it from the north winds, while the land slopes towards the south making drainage an easy matter. The Sanatorium was opened in 1899. It is two stories in height, except the central part which runs up to three. This third story improves the appearance of the building and it provides accommodation for some of the staff.

The ground floor has a large dining-room, a small drawing-room and rooms for seven patients. On the first floor are rooms for 13 patients. The bath-rooms and sanitary annexes are to the north, and the closets are properly cut off from the main building by cross-ventilated lobbies. In addition to the Sanatorium proper there are three châlets and a bungalow; and the total accommodation is for 34 patients. Further there is a cottage on the grounds in which patients' friends can be received at a fixed charge per diem.

To us the whole of the arrangements seemed to be of a most satisfactory nature. There are many "shelters" provided in the gardens, and pleasant walks have been cut through a wood. Vegetables are grown on the estate and milk is supplied by the dairy of the Sanatorium. The water is pumped from a deep well on the grounds and the Sanatorium is lighted by electricity. The rector of the parish holds a service for the patients on Sunday afternoons. The hours for meals are—breakfast at half-past eight, luncheon at half-past one, and dinner at seven. It need hardly be said that the diet is liberal. The staff consists of four nurses, ten domestic servants, and four men-servants. The terms for patients range from four to five guineas a week, which sums include everything except personal laundry, stimulants, and special nursing should it ever be required. The duration of the treatment can hardly be definitely stated, but approximately it is from four to six months.

Records of cases treated are carefully kept by Dr. Noel Bardswell, the resident physician, and it would seem that in this establishment about 60 per cent. of the patients are cured of the disease and that a high proportion of the others have been improved. An important point is that Dr. Bardswell does not lose sight of his patients after they leave the sanatorium, and we learn that 93 per cent. of those in whom the disease was arrested are still living, and that at least 77 per cent. have maintained their health and are able to follow their employment. Such facts are alike gratifying to the patient, the physician, and the advocate of this rational method of treating phthisis.

